# Inflammatory Modulation of miR-155 Inhibits Doxorubicin-Induced Testicular Dysfunction via SIRT1/FOXO1 Pathway: Insight into the Role of Acacetin and *Bacillus cereus* Protease

**DOI:** 10.1007/s12010-022-03992-8

**Published:** 2022-06-18

**Authors:** Hend Mohamed Anwar, Sherin Ramadan Hamad, Gad Elsayed Mohamed Salem, Rania Hassan Mohamed Soliman, Eman Maher Elbaz

**Affiliations:** 1 Department of Biochemistry, National Organization for Drug Control & Research, Giza, 11221 Egypt; 2 Department of Histopathology, National Organization for Drug Control & Research, Giza, 11221 Egypt; 3 Department of Microbiology, National Organization for Drug Control & Research, Giza, 11221 Egypt; 4 Reef Biology Research Group, Department of Marine Science, Faculty of Science, Chulalongkorn University, Bangkok, 10700 Thailand; 5 Department of Anatomy and Embryology, Faculty of Medicine, Zagazig University, Zagazig, Egypt; 6 Department of Biochemistry, Faculty of Pharmacy, Cairo University, Kasr El Aini St, Cairo, 11562 Egypt

**Keywords:** Doxorubicin, miR-155, SIRT1, FOXO1, Acacetin, *Bacillus cereus* protease

## Abstract

Doxorubicin (DOX) is a chemotherapeutic agent that can disrupt testicular function leading to male infertility. This study examined the protective role of natural flavone, acacetin (ACA), and a protease of *Bacillus cereus* bacteria (*B. cereus*) as well as the potential role of miR-155/SIRT1/FOXO1 network in DOX-induced testicular injury. Twenty-four male Wistar rats were randomly allocated into four groups and treated as follows: Control, DOX (1 mg/kg, i.p) every other day for 21 days with a total dose equal to 10 mg/kg throughout the experiment, and pre-treated groups that received ACA (5 mg/kg/day, p.o) or *B. cereus* protease (36 mg/kg/day, p.o) for a week prior to DOX administration. DOX challenge reduced the testis weight coefficient, serum testosterone, and testicular 17β-hydroxysteroid dehydrogenase (17β-HSD). DOX caused a significant increase in testicular oxidative stress, inflammatory, and apoptotic markers. Aberrant testicular miR-34c, a germ-specific miRNA, and miR-155 expressions were observed, along with decreased protein expression of sirtuin1 (SIRT1) dependent forkhead box 1 (FOXO1) acetylation which induces apoptosis. Besides, abnormal histopathological architecture and a marked reduction in the testicular expression of proliferating cell nuclear antigen (PCNA) were observed. ACA or protease administration significantly improved the histopathological and immunohistochemical pictures compared with DOX alone and renovated testicular functions. Interestingly, treatment with protease was more significant than treatment with ACA in ameliorating DOX-induced testicular injury. Taken together, this study reveals the prophylactic role of these two regimens on male fertility by exhibiting antioxidant, anti-inflammatory, and anti-apoptotic effects against DOX-elicited testicular damage, possibly via modulating miR-155/SIRT1/FOXO1 network.

**Graphical abstract:**

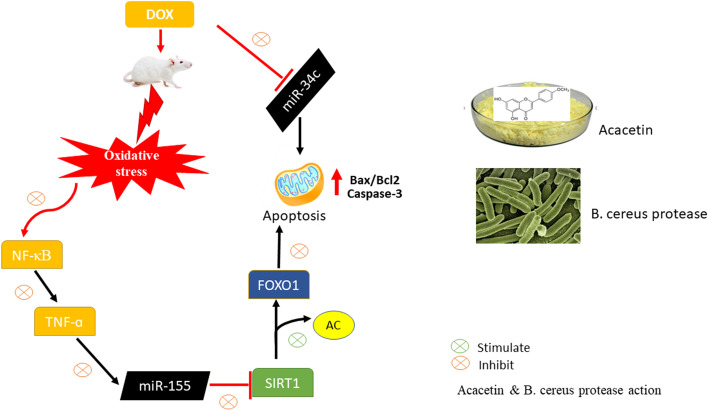

**Supplementary Information:**

The online version contains supplementary material available at 10.1007/s12010-022-03992-8.

## Introduction

Doxorubicin (DOX) is an anthracycline-derived antibiotic that is frequently used to treat a variety of tumor types [1]. Its effects are not only limited to cancer cells; but DOX can also harm healthy cells, particularly those that exhibit rapid and continuous proliferative activity such as male sperm cells [2]. DOX can impair spermatogenesis, trigger testicular damage, and eventually cause male infertility [3]. Noteworthy, the majority of patients treated with DOX were azoospermic, whereas one-third of them were still azoospermic 5 years afterward [4].

Oxidative stress, lipid peroxidation, and apoptosis are considered the main mechanisms responsible for DOX-induced testicular injury [5, 6]. DOX affects testicular integrity throughout both the prepubertal and postpubertal phases of development [7].

MicroRNAs (miRNAs) are non-coding RNAs of 19–25 nucleotides that play important roles in a variety of biological processes [8]. They have the potential as diagnostic markers and therapeutic targets in a variety of diseases [9]. Some miRNAs are elevated during the oxidative stress and inflammatory responses and contribute to degenerative diseases [10]. A well-studied miRNA, miR-155, is directly regulated by pro-inflammatory reactions [11], and miR-155 overexpression has been linked to nuclear factor-kappa B (NF-κB) transcriptional regulation, which is triggered by the inflammatory cytokine tumor necrosis factor-alpha (TNF-α) [9]. In addition, miR-155 upregulation was reported to inhibit the SIRT1 (sirutin1) pathway [12]. Moreover, miR-34c, a member of miR-34 family: miR23a-miR34b-and miR34c, is abundantly expressed in the testis [13, 14]. It has a role in spermatogenesis, and its downregulation causes infertility in male mice [15]. miR-34c is markedly decreased in both testicular tissues of patients with cryptorchidism and in a murine model of cryptorchidism [16].

SIRT1 is a NAD-dependent deacetylase that can deacetylate multiple transcriptional factors, including forkhead box O (FOXO), NF-κB, and the mitochondrial biogenesis coactivator PGC-1alpha [17–19]. It plays a crucial role in cell proliferation and differentiation [20]. In vitro studies indicate that SIRT1 suppresses the activity of Bcl-2-associated X protein (Bax), FOXO, and Rb (retinoblastoma) [21, 22]. SIRT1 was reported to protect b-cells from oxidative stress via a mechanism that involves the deacetylation of FOXO proteins [23].

FOXO transcription factors regulate several aspects of development, metabolism, and reproduction [24]. There are four members of the FOXO family: including FOXO1, FOXO2, FOXO3, and FOXO4 [25]. The FOXO protein family is broadly responsible for signal transduction, growth and development, apoptosis, and oxidative stress, of which FOXO1 and FOXO3 are the most prevalent [26]. Only three members of this family: FOXO1, FOXO3, and FOXO4 have been identified in humans [27]. FOXO1 plays an important role in the male germline [28], where it is expressed particularly in undifferentiated spermatogonia cells, which act as a stem cell population that drives spermatogenesis [28]. SIRT1 and FOXO interact in a complex manner to protect against oxidative damage [29]. SIRT1 binds to FOXO1, inhibits its acetylation, and reduces its transcriptional activity [30]. Cigarette smoke has been demonstrated to produce oxidative stress injury in lung cells by acting on the SIRT1/FOXO pathway [31]. Upon activation of the SIRT1/FOXO pathway, the degree of FOXO deacetylation not only regulates oxidative stress but also controls cell apoptosis and the cell cycle, in a complex and interactive process [30]. Therefore, studies on the role of this pathway in the injury of toxins warrant further study.

Acacetin (ACA): 5,7-dihydroxy-4′-methoxyflavone is a natural flavone that occurs in a variety of plant pigments. It exhibits anti-oxidative, anti-inflammatory, and anti-apoptotic effects [32]. ACA is effective for treating doxorubicin cardiomyopathy by enhancing AMPK/Nrf2 antioxidative signaling molecules [1]. It protects the myocardium from ischemia/reperfusion and inhibits apoptosis of H9c2 cardiomyocytes through the PI3K/Akt pathway [33]. Although ACA has medicinal benefits, its effects on DOX-induced testicular damage are unknown.

Proteases are a new class of therapeutics with significant potential. In the human genome, more than 2% of the genes encode for proteases [34]. For example, proteases modulate growth factors, cytokines, chemokines, and cell receptors, to affect gene regulation and downstream intracellular signaling [35]. The US FDA has approved a variety of proteases for therapeutic applications. For example: Tissue-type plasminogen activator (t-PA) and factor IX (FIX) are proteases used in the management of cardiovascular diseases such as stroke, acute myocardial infarction, and bleeding in patients with hemophilia, respectively [35]. Single orally administered proteolytic enzymes of plant and animal origin are widely used as a treatment for a variety of digestive, absorptive, and pancreatic disorders. Porcine and bovine pancreatic enzymes are the preferred form of supplementation for exocrine pancreatic insufficiency [35]. Plant-based enzymes, such as bromelain from pineapple, are also effective as digestive aids for the breakdown of proteins [36]. However, orally administered proteolytic enzyme combinations often supplemented with rutosid are widely used as an alternative or a supplementary treatment for various syndromes, such as acute and post-surgical trauma, phlebitis, rheumatoid arthritis, osteoarthritis, and as adjunctive therapy for cancer [37, 38].

Although proteases are synthesized by different plants, animals, and microorganisms, the latter are the most common in nature. Proteases have potential as anti-inflammatory agents. They have been shown to work in harmony with non-steroidal anti-inflammatory (NSAIDs) drugs, either independently or synergistically. However, the use of NSAIDs has negative side effects such as hepatorenal damage [39, 40], sperm cell toxicity, and testicular dysfunction in rats [41]. Therefore, using bioactives and enzymes with anti-inflammatory action may assist in reducing the use of NSAIDs [42]. *Bacillus cereus* is a type of gram-positive bacteria, which produces an alkaline protease commonly found in soil and exhibited high proteolytic activity. *B. cereus* produces an enzyme that possesses fibrinolytic potential in vitro [43].

Therefore, in the current study, we evaluated the protective effects of ACA or *B. cereus* protease against DOX-induced male infertility in rats and investigated the role of miR-155/SIRT1/FOXO1 signaling pathway.

## Material and Methods

### Animals

Adult male Wistar albino rats, weighing 150–170 g, were obtained from the National Organization for Drug Control and Research (NODCAR), Giza, Egypt. Rats were housed in stainless steel cages under controlled environmental conditions: temperature (23 °C ± 2 °C), humidity (60% ± 10%), ventilation 10–20 changes/h, and a 12 h/12 h light/dark cycle at the animal house facility of NODCAR, Giza, Egypt. The rats were fed a standard chow diet and allowed water ad libitum. The investigation complied with the Guide for the Care and Use of Laboratory Animals and was approved by the Ethics Committee for Animal Experimentation at Faculty of Pharmacy, Cairo University (Permit Number: BC 3114).

### Drugs and Chemicals

DOX was obtained from Novartis Pharmaceutical Co. (El Amireya, Cairo, Egypt). ACA was purchased from Sigma-Aldrich (St. Louis, MO, USA) and diluted in dimethyl sulfoxide (DMSO) (Sigma-Aldrich, St. Louis, MO, USA). Protease *B. cereus* strain was isolated and purified as described below. All chemicals were of the highest purity and analytical grade.

### Microorganism and Enzyme Production

The bacterial culture, *B. cereus* S6-3 was isolated from soil samples collected in Egypt’s Sharkia governorate. The molecular identification and optimization of fermentation parameters for optimum enzyme production were previously reported [44]. The medium used for the production of enzyme by parent and mutant strains consisted of (g/l): skimmed milk (6), fructose (10), K_2_HPO_4_ (0.5), yeast extract (1), MgSO_4_·7H_2_O (1), KCl (5), CaCl_2_·2H_2_O (0.2), and NaCl (5), at a final pH of 6.0 [44]. The selected mutant strain *B. cereus* S6-3/UM90, used for protease production, was described previously [45].

### Partial Purification of Protease Enzyme

After centrifugation, the clear supernatant from the culture media was precipitated with acetone [46]. Briefly, the cell-free supernatant and cooled acetone (− 20 °C) were combined at a 1:2 ratio and centrifuged for 10 min at 10,000 rpm. The precipitate was collected and air-dried at room temperature. The dried pellet was re-suspended in a minimum volume of 10 mM phosphate buffer, pH 7.5. Partial purification of protease produced from mutant *B. cereus*-S6-3/UM90 is shown in Table 1. The enzyme solution was first precipitated with saturated acetone, which increased the protease activity by 1.72 fold with a 62% recovery, exhibiting a specific activity of 186 U/mg [47, 48].

**Table 1:** Summary of partial purification of protease obtained from *Bacillus cereus*-S6-3/UM90

Purification steps	Volume (mL)	Total activity (U)	Total protein (mg)	Specific activity (U*/*mg)	Yield or recovery (%)	Fold purification
Crude	100	42,900	395	108	100	1
Acetone-treated	100	26,598	143	186	62	1.72

### Estimation of Enzymatic Activity

The proteolytic activity was measured according to Kembhavi et al. [49] method with some minor modifications. Briefly, 5.0 ml of (1.0%, w/v) casein (substrate) was prepared in 10 mM carbonate-bicarbonate buffer-pH-10.5. A total of 1.0 ml of the supernatant solution was added to the substrate and incubated at 40 °C for 10 min. A blank test tube was incubated without the addition of enzyme solution. The enzymatic reaction was stopped by adding 5.0 ml of 0.4 M trichloroacetic acid solution. The reaction mixtures were allowed to stand for 25 min at room temperature. Then, the solutions were centrifuged at 5000 rpm for 10 min to remove the precipitate. The absorbance of the clear supernatant was measured at 660 nm. Tyrosine (0–50 mg/ml) calibration curve was used as a standard calibration curve. One protease activity unit was defined as the amount of enzyme required to liberate 1 mg of tyrosine/ml/min under the standard experimental conditions.

### Experimental Design

In the current study, 24 rats were randomly allocated into four groups (6 rats each), as follows:

The first group received 1% dimethyl sulfoxide (DMSO) (1 ml/kg/day) orally for 28 days plus phosphate-buffered saline (PBS) (1 ml/kg) every other day for 21 days (starting from day 8) and served as a control group.

The second group received DOX (1 mg/kg/i.p) in PBS every other day for 21 days, resulting in a total of 10 mg/kg throughout the experimental period [50] and served as a paradigm for reproductive damage. DOX was prepared in PBS as a stock solution at 1 mg/ mL (i.p volume 1 ml/kg).

The third group received ACA (5 mg/kg/day p.o.) diluted in 1% DMSO for 1 week [51] then received DOX as in the second group concurrently with ACA (5 mg/kg/day p.o.) for 21 days. ACA was prepared in 1% DMSO as a stock solution at 5 mg/ mL (p.o. volume 1 ml/kg).

The fourth group received bacterial protease (36 mg/kg/day, p.o.) in PBS for 1 week [52] then received DOX as in the second group concurrently with bacterial protease (36 mg/kg/day, p.o.) for 21 days. Protease was prepared in PBS as a stock solution at 36 mg/ mL (i.p volume 1 ml/kg).

Animal body weight was recorded weekly throughout the study. 24 h after the end of the experiment, 2 ml of blood was withdrawn from the retro-orbital plexus vein under light anesthesia (thiopental sodium 5 mg/kg, i.p) [53]. Sera were separated for the measurement of serum testosterone levels. After that, the animals were euthanized. Both testes were immediately dissected out, washed with ice-cold saline, dried, and weighed. For each rat, the organ coefficient (testes weight/body weight) was calculated. A tissue portion was fixed in 10% formalin for histopathological examination. Another part was homogenized in ice-cold-buffered saline (1:9 w/v) for measuring 17β-hydroxysteroid dehydrogenase (17β-HSD), SIRT1, FOXO1, nitric oxide (NO), and oxidative stress markers. The remaining testicular tissue was kept at − 80 °C for gene expression analysis.

### Biochemical Analysis

#### Testicular Damage Markers

Serum testosterone levels were measured using an enzyme-linked immunosorbent assay kit (ELISA) supplied by Diametra (Perugia, Italy, Ref DK0015). 17β-HSD protein expression levels were determined in tissue homogenate with an ELISA kit obtained from MyBioSource, Inc. (San Diego, USA, Cat.No. MBS2104946) according to the manufacturer’s instructions. The results are expressed as pg/ml for serum testosterone and ng/g tissue for 17β-HSD.

#### Testicular Redox State

Tissue-reduced glutathione (GSH) and malondialdehyde (MDA) levels were determined as previously described [54, 55]. The results are expressed as μmol/g tissue for GSH and nmol/g tissue for MDA. NO levels, superoxide dismutase (SOD) activity, and total antioxidant capacity (TAC) were measured using NO, SOD, and TAC kits, from Biodiagnostics, Egypt, (Cat. No. NO 25 33, SD 25 21, and TA 25 13, respectively). The results are expressed as μmol/L for NO, U/g tissue for SOD, and mM/L for TAC.

#### qRT-PCR Analysis of Nrf2, TLR4, NF-κB, Bax, and Bcl2 Expression

Samples were stored in RNA lysis solution at − 80 °C. The expression of Nrf2, TLR4, NF-κB, Bax, and Bcl2 mRNA was assessed by real-time quantitative reverse transcription PCR (RT-PCR) using standard protocols. The total RNA was converted into complementary DNA (cDNA) using ExcelRTTM Reverse Transcription Kit (SAMOBIO, Small Bio Smart tool, Cat. No. RP1300). Real-time PCR was conducted using a DTlite real-time PCR System (DTlite, DNA technology, LLC, Moscow, Russia) and BioEasy SYBR Green Master Mix (Bioer Technology, Cat. No. BSB25L1) in a final volume of 25 µl. Thermal cycling conditions included 95 °C for 15 s, followed by 40 cycles at 95 °C for 15 s, 60 °C for 15 s, and 72 °C for 45 s. The data were analyzed using ABI Prism software and quantified using PE Biosystems v1_7 Sequence Detection Software (Foster City, CA, USA). Using the comparative threshold cycle method, we calculated the relative expression of the genes. All values were normalized to the expression of an endogenous control gene (GAPDH) as an invariant control. Primer sequences for Nrf2, TLR4, NF-κB, Bax, and Bcl2 are listed in Table 2.

**Table 2:** Primers sequence used in the experiment

Name of primer	Sequence	Accession No
Nrf2	Forward 5′CAAATCCCACCTTGAACACA 3′ Reverse 5′CGACTGACTAATGGCAGCAG 3′	XM_032903520.1
TLR4	Forward 5′-CACTGTTCTTCTCCTGCCTGAC-3′ Reverse 5′-TGG TTGAAGAAGGAATGTCATC-3′	NM_021297.3
NF-κB	Forward 5′-CCT CTGGCGAATGGCTTTAC-3′ Reverse 5′-GCTATGGAT CTGCGGTCTGG-3′	NM_009045.5
Bax	Forward 5′-GAACCATCATGGGCTGGACA-3′ Reverse 5′-TGAGGTTTATTGGCGCCTCC-3′	XM_032915032.1
Bcl2	Forward 5′-GAACTGGGGGAGGATTGTGG-3′ Reverse 5′-ACTTCACTTGTGGCCCAGAT-3′	XM_034943915.1
GAPDH	Forward 5′-ACCACAGTCCATGCCATCAC-3′ Reverse 5′-GTCCTCAGTGTAGCCCAGGA-3′	XM_034500817.1
miR-34c	Forward 5′-AGTTACTAGGCAGTGTAG-3′ Reverse 5′-TCTTTTTACCTGGCCGTGT-3′	XM_017596027.2
miR-155	Forward 5′-TAATGCTAATCGTGATAGGGGTT-3′ Reverse 5′- CACCGTACCCTGTTAATGCT-3′	NR_129131.2
RNU6B	Forward 5′-CTCGCTTCGGCAGCACA-3′ Reverse 5′-AACGCTTCACGAATTTGCGT-3′	XR_003233292.1

#### Testicular miR-34c and miR-155

TRIzol® reagent (Invitrogen, Sigma-Aldrich, St. Louis, MO, USA) was used to extract the total RNA from frozen samples. For the evaluation of miR-34c and miR-155, the miRNeasy extraction kit (Qiagen, Cat. No. / ID: 217,084) was used. By standard protocols, the total RNA was converted into complementary DNA (cDNA) using ExcelRTTM Reverse Transcription Kit (SAMOBIO, Small Bio Smart tool, Cat. No. RP1300). The real-time PCR was conducted using a DTlite real-time PCR System (DTlite, DNA technology, LLC, Moscow, Russia) and BioEasy SYBR Green Master Mix (Bioer Technology, Cat. No. BSB25L1) in a final volume of 25 µl. Thermal cycling conditions included 95 °C for 15 s, followed by 40 cycles at 95 °C for 15 s, 60 °C for 15 s, and 72 °C for 45 s. Changes in the expression of each miRNA were normalized to the endogenous control gene RNU6B. Relative expression was calculated by 2 − ΔCt in each group. Primer sequences for miR-34c and miR-155 are listed in Table 2.

#### Testicular SIRT1 and FOXO1

SIRT1 and FOXO1 levels were estimated using ELISA kits provided by MyBioSource, Inc. (San Diego, USA) (Cat.No. MBS060720, and MBS749342, respectively) according to the manufacturer’s instructions, and the results are expressed as ng/g tissue.

#### Histopathological Examination

Testes were fixed in 10% formalin for 24 h. Pieces of testes were dehydrated in increasing concentrations of alcohol and cleaned in. After that, the samples were embedded in paraffin wax. Five-micrometer-thick sections were deparaffinized with xylene and stained with hematoxylin and eosin (H&E). The slides were analyzed by light microscopy, and photomicrographs were captured at a power of × 200.

#### Immunohistochemistry

Four-micrometer-thick sections of testis samples were placed into a pressure cooker containing Tris–EDTA buffer with 0.05% tween 20 (pH 9.0), for 3 min for antigen retrieval, followed by blocking of endogenous peroxidase with 3% hydrogen peroxide in phosphate-buffered saline for 5 min, then washing with distilled water and Tris-buffered saline containing 0.05% tween 20 (TBST, pH 8.4). Thereafter, sections were incubated with polyclonal primary antibodies for TNF-α (1:120)  Cat. No. A356015, caspase-3 (1:150) Cat. No. PK-CA577-K16, and PCNA (1:50) Cat. No. OKCD02760 (Cloud-Clone Corp, USA) for 24 h at 4 °C. After washing with TBST, sections were incubated with Dako EnVision™ + System/HRP-labeled polymer containing goat anti-rabbit secondary antibody (Agilent Technologies, Inc. USA) for 30 min at room temperature. Visualization was performed using Dako 3,3′-diaminobenzidine substrate (Agilent Technologies, Inc. USA) for 5 min at room temperature. Sections were counter-stained in hematoxylin for 5 s, dehydrated, and viewed using a light microscope (Olympus BX41, UK). Quantitative measurement of the percentage area of TNF-, as well as caspase-3 and PCNA immunostaining color intensity, was done by analyzing the intensity of the brown stain in the image using ImageJ software. (ImageJ, NIH-Bethesda, MD, USA).

#### Statistical Analysis

The data are presented as the mean ± standard deviation (SD), with a one‐way analysis of variance (ANOVA) followed by Tukey’s post-hoc test. Moreover, associations between different parameters were assessed using Pearson correlation analysis. GraphPad Prism software (version 8; GraphPad Software, Inc., San Diego, CA, USA) was used for statistical analyses and presenting the data. The level of significance *P* < 0.05 was fixed for all statistical tests.

## Results

### Effect of ACA or B. cereus protease on the Testis Organ Coefficient, Serum Testosterone, and Testicular 17β-HSD in DOX-Induced Testicular Damage in Rats

**Fig. 1:**
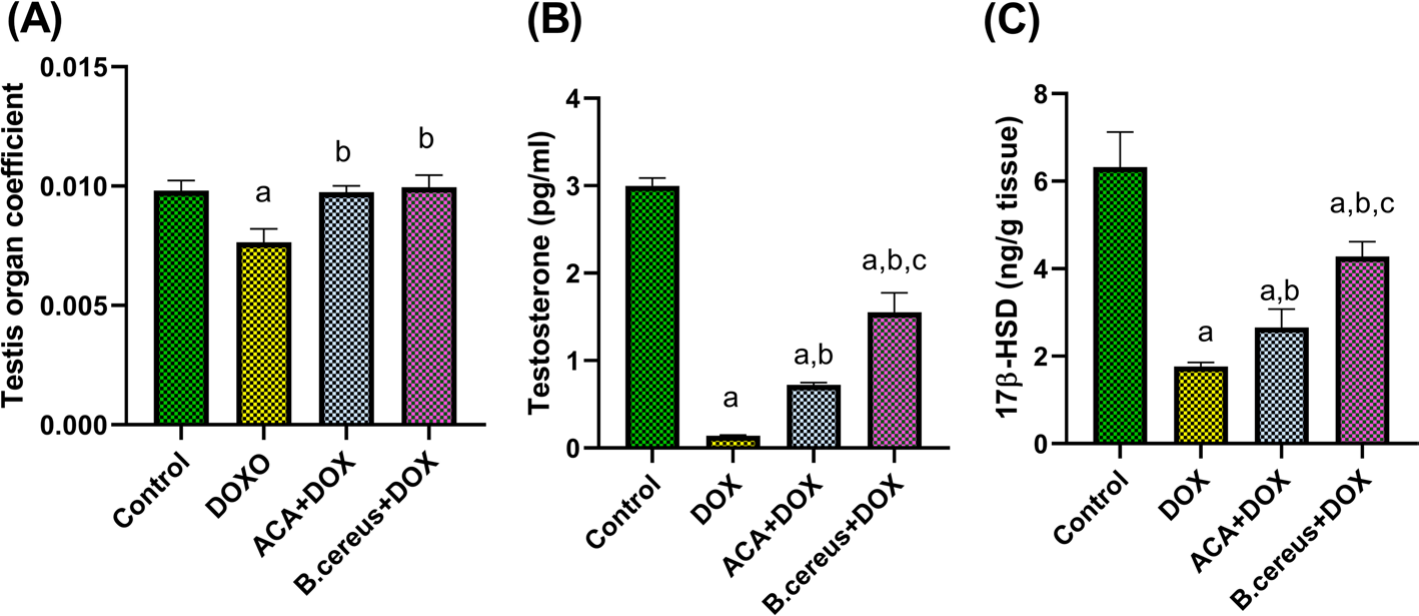
Effect of ACA and *B. cereus* protease on the testis organ coefficient (**A**), serum testosterone levels (**B**), and testicular 17β-HSD (**C**) in DOX-induced testicular damage in rats: Each bar with a vertical line represents the mean ± S.D (*n* = 6). ^a^Compared with the control group, ^b^compared with the DOX group, and ^c^compared with the ACA + DOX group. All values are statistically significant at *P* < 0.05. Abbreviations: ACA, acacetin; *B. cereus*, *Bacillus cereus* protease; 17β-HSD, 17 beta-hydroxysteroid dehydrogenases; DOX, doxorubicin

As depicted in Fig. 1A, DOX administration significantly reduced the testis organ coefficient by 22% compared with the control (*P* < 0.0001). On the other hand, treatment with ACA or *B. cereus* normalized the testis organ coefficient. Therefore, ACA or *B. cereus* may preserve testicular growth and development. Moreover, DOX-induced a significant decrease in serum testosterone levels (Fig. 1B), and tissue 17β-HSD (Fig. 1C) by 93% and 68%, respectively, compared with the control group (*P* < 0.0001). However, treatment with ACA or *B. cereus* induced a significant increase in the aforementioned parameters compared with the rats treated with DOX alone.

### Effect of ACA and B. cereus Protease on Oxidative Stress Biomarkers in DOX-Induced Testicular Damage in Rats

**Fig. 2:**
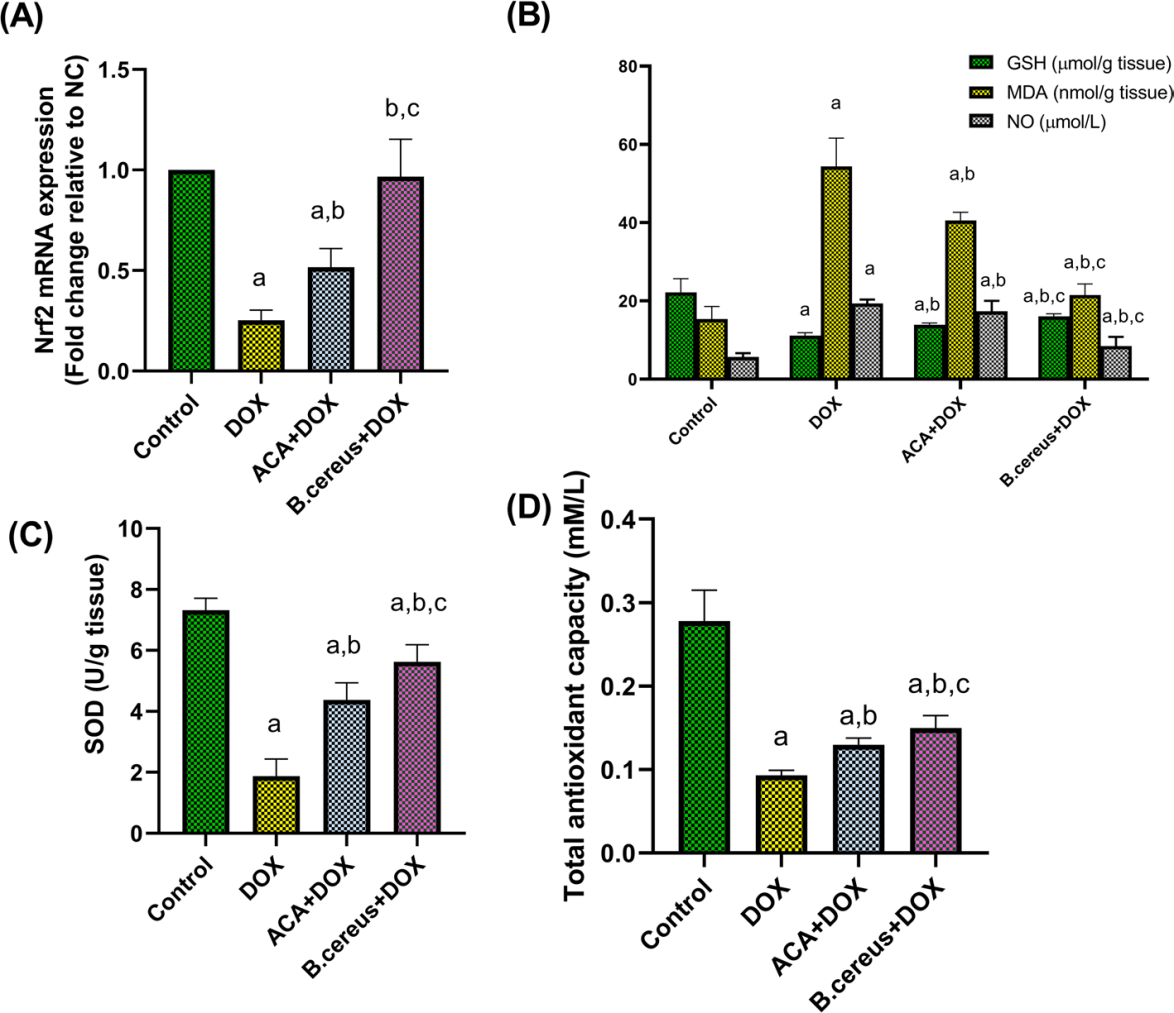
Effect of ACA and *B. cereus* protease on Nrf2 gene expression (**A**), GSH; MDA; and NO levels (**B**), SOD activity (**C**), and TAC (**D**) in DOX-induced testicular damage in rats: Each bar with a vertical line represents the mean ± S.D (*n* = 6). ^a^Compared with the control group, ^b^compared with the DOX group, and ^c^compared with the ACA + DOX group. All values are statistically significant at *P* < 0.05. Abbreviations: ACA, acacetin; *B. cereus*, *Bacillus cereus* protease; DOX, doxorubicin; GSH, reduced glutathione; MDA, malondialdehyde; NC, normal control; Nrf2, the nuclear factor erythroid 2-related factor 2; NO, nitric oxide; SOD, superoxide dismutase; TAC, total antioxidant capacity

DOX significantly increased MDA level, and NO content in testis by threefold and fourfold, respectively, when compared with the control (*P* = 0.009, and *P* < 0.0001, respectively), whereas pre-treatment with ACA markedly reduced MDA level and NO content (*P* = 0.03). Pretreatment with *B. cereus* protease significantly decreased MDA level (*P* = 0.0004) and NO content (*P* < 0.0001) (Fig. 2B). Similarly, Nrf2 gene expression, GSH, SOD, and TAC levels were significantly lowered in the testicular tissues of the DOX-only-treated group by 70%, 50%, 73%, and 64%, respectively, compared with that of the control (Fig. 2A, 2B, 2C, and 2D). However, pre-treatment with ACA or B. cereus protease significantly improved the antioxidant parameters. In addition to normalization of Nrf2 gene expression level in B. cereus-treated group (*P* = 0.97), B. cereus protease group exhibited a greater ameliorative effect in the abovementioned parameters (*P* = 0.0014, < 0.0001, 0.001, 0.0008, respectively) compared with ACA treatment (*P* = 0.023, 0.0052, 0.001, 0.028, respectively).

### Effect of ACA and B. cereus Protease on Inflammatory Biomarkers in DOX-Induced Testicular Damage in Rats

**Fig. 3:**
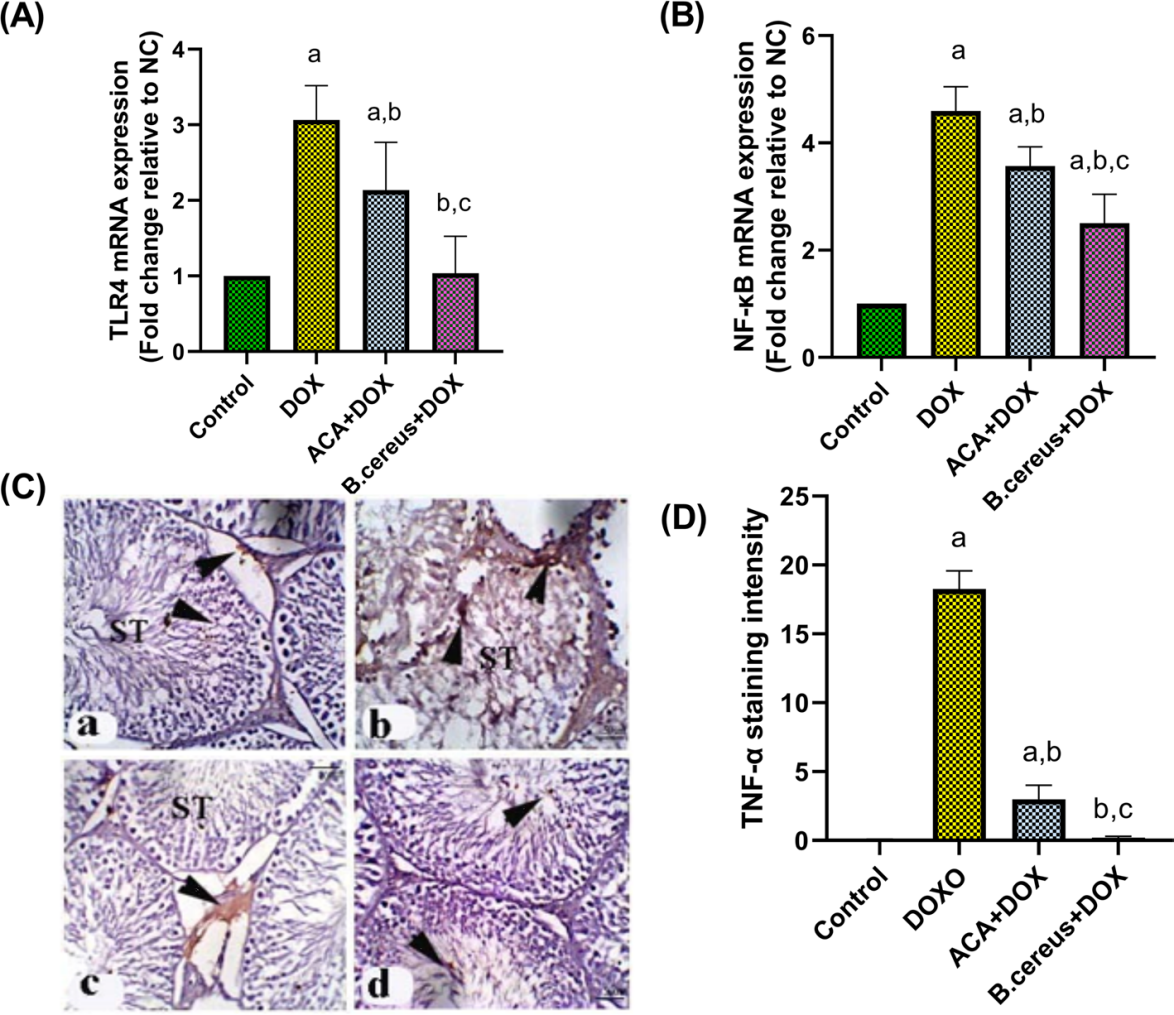
Effect of ACA and *B. cereus* protease on inflammatory biomarkers; TLR4 (A), NF-κB (B) gene expressions, and TNF- (C) protein expression in DOX-induced testicular damage in rats: Each bar with a vertical line represents the mean ± S.D (*n* = 6). Control group (**a**); DOX-treated group (**b**); DOX + ACA–treated group (**c**); and DOX + *B. cereus* protease–treated group (**d**). X 200 (Scale bar = 50 μm). ^a^Compared with the control group, ^b^compared with the DOX group, and ^c^compared with the ACA + DOX group. All values are statistically significant at *P* < 0.05. Abbreviations: ACA, acacetin; *B. cereus*, *Bacillus cereus* protease; DOX, doxorubicin; NC, normal control; NF-κB, nuclear factor kappa-light-chain-enhancer of activated B cells; TLR4, toll-like receptor 4; TNF-α, tumor necrosis factor-alpha

DOX significantly upregulated the testicular gene expression of TLR4, and NF-κB by threefold and 4.5-fold, respectively, compared with the control group (*P* = 0.0004 and *P* < 0.0001, respectively) (Fig. 3A and 3B). Moreover, DOX showed positive immunostaining for TNF-α (*P* < 0.0001) (Fig. 3C) compared with the control group. Nevertheless, pre-treatment with ACA or *B. cereus* protease significantly halted the upregulation of TLR4, and TNF-α (*P* = 0.0002 and *P* < 0.0001, respectively). However, the protease-treated group showed a more pronounced inhibitory effect on NF-κB expression (*P* < 0.0001) than ACA-treated group (*P* = 0.01) against DOX-induced testicular damage.

### Effect of ACA and B. cereus Protease on Testicular miR-34c and miR-155 Gene Expression Levels in DOX-Induced Testicular Damage in Rats

**Fig. 4:**
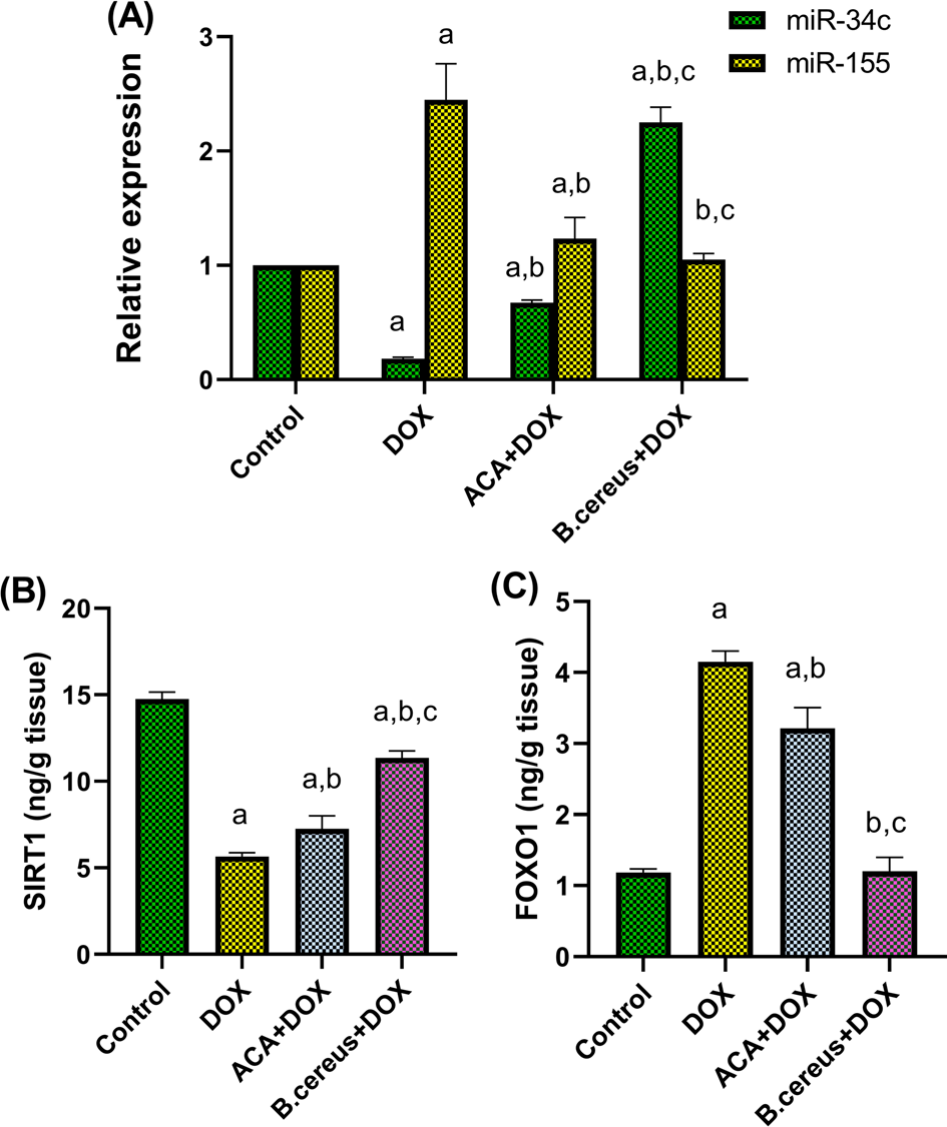
Effect of ACA and *B.* cereus protease on testicular miR-34c and miR-155 gene expressions (**A**), testicular SIRT1 (**B**), and FOXO1 (**C**) protein expressions in DOX-induced testicular damage in rats: Each bar with a vertical line represents the mean ± S.D (*n* = 6). ^a^Compared with the control group, ^b^compared with the DOX group, and ^c^compared with the ACA + DOX group. All values are statistically significant at *P* < 0.05. Abbreviations: ACA, acacetin; *B. cereus*, *Bacillus cereus* protease; DOX, doxorubicin; FOXO1, Forkhead box protein O1; miR, microRNA; SIRT1, sirtuin-1

DOX-treated rats induced a significant downregulation of miR-34c expression level by 80% and a significant upregulation of miR-155 expression by 2.5-fold when compared with the control group. In contrast, pretreatment with either ACA or *B. cereus* protease significantly reduced the changes observed in miR-34c (*P* < 0.0001), and miR-155 (*P* = 0.0053 and *P* = 0.0003, respectively) expression levels (Fig. 4A).

### Effect of ACA and B. cereus Protease on Testicular SIRT1/FOXO1 Protein Expression Levels in DOX-Induced Testicular Damage in Rats

DOX-treated group depicted a significant reduction in SIRT1 protein expression by 66% (Fig. 4B) and a subsequent significant increase in FOXO1 protein expression by fourfold (Fig. 4C) when compared with the control group (*P* < 0.0001). These impairments were mitigated in ACA and the bacterial protease–treated groups (*P* < 0.0001).

### Effect of ACA and B. cereus Protease on Apoptotic Biomarkers in DOX-Induced Testicular Damage in Rats

**Fig. 5:**
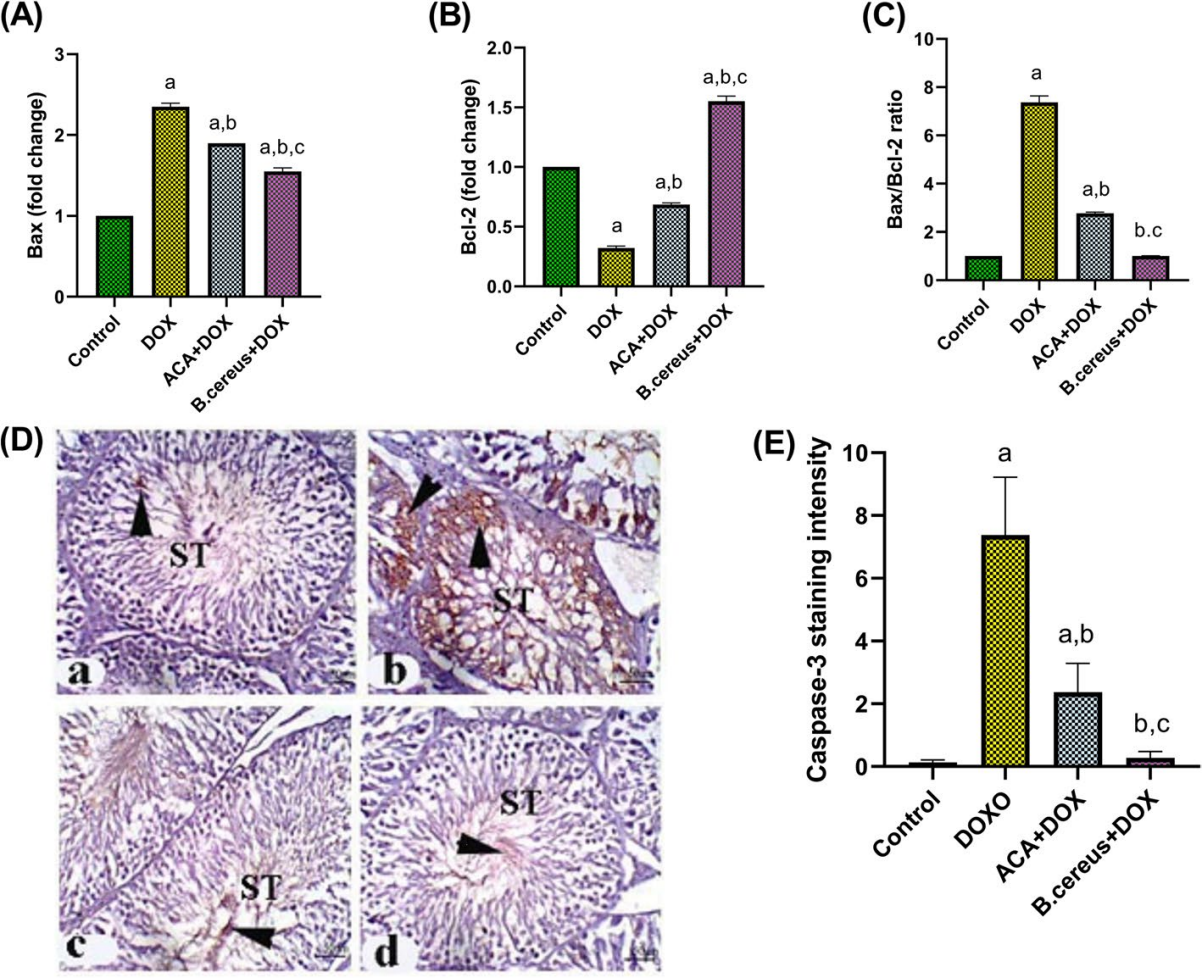
Effect of ACA and *B. cereus* protease on Bax gene expression (**A**), Bcl2 gene expression (**B**), Bax/Bcl2 ratio (**C**), Caspase-3 protein level (**D**), and Caspase-3 staining intensity (**E**) in DOX-induced testicular damage in rats: Each bar with a vertical line represents the mean ± S.D (*n* = 6). Control group (**a**); DOX-treated group (**b**); DOX + ACA–treated group (**c**); and DOX + *B. cereus* protease–treated group (**d**). X 200 (Scale bar = 50 μm). Arrow: Brown color is a positive reaction of the tested marker. ^a^Compared with the control group, ^b^compared with the DOX group, and ^c^compared with the ACA + DOX group. All values are statistically significant at *P* < 0.05. Abbreviations: ACA, acacetin; *B. cereus*, *Bacillus cereus* protease; Bax, Bcl-2-associated X protein; Bcl2, B-cell lymphoma 2; DOX, doxorubicin; ST, seminiferous tubules

As demonstrated in Fig. 5C, a significant increase was observed in the Bax/Bcl2 ratio of the DOX-intoxicated group as compared with the control group (*P* < 0.0001). However, ACA-treated group showed a significant reduction in the Bax/Bcl2 ratio as compared with the DOX-treated group (*P* < 0.0001). Whereas the bacterial protease–treated group restored this ratio to the normal level (*P* = 0.46). DOX group showed significant positive staining for caspase-3 as compared with the control group (Fig. 5E). While ACA and bacterial protease–treated groups showed a significant reduction in caspase-3 staining intensity as compared with the DOX-treated rats (*P* < 0.0001).

**Fig. 6:**
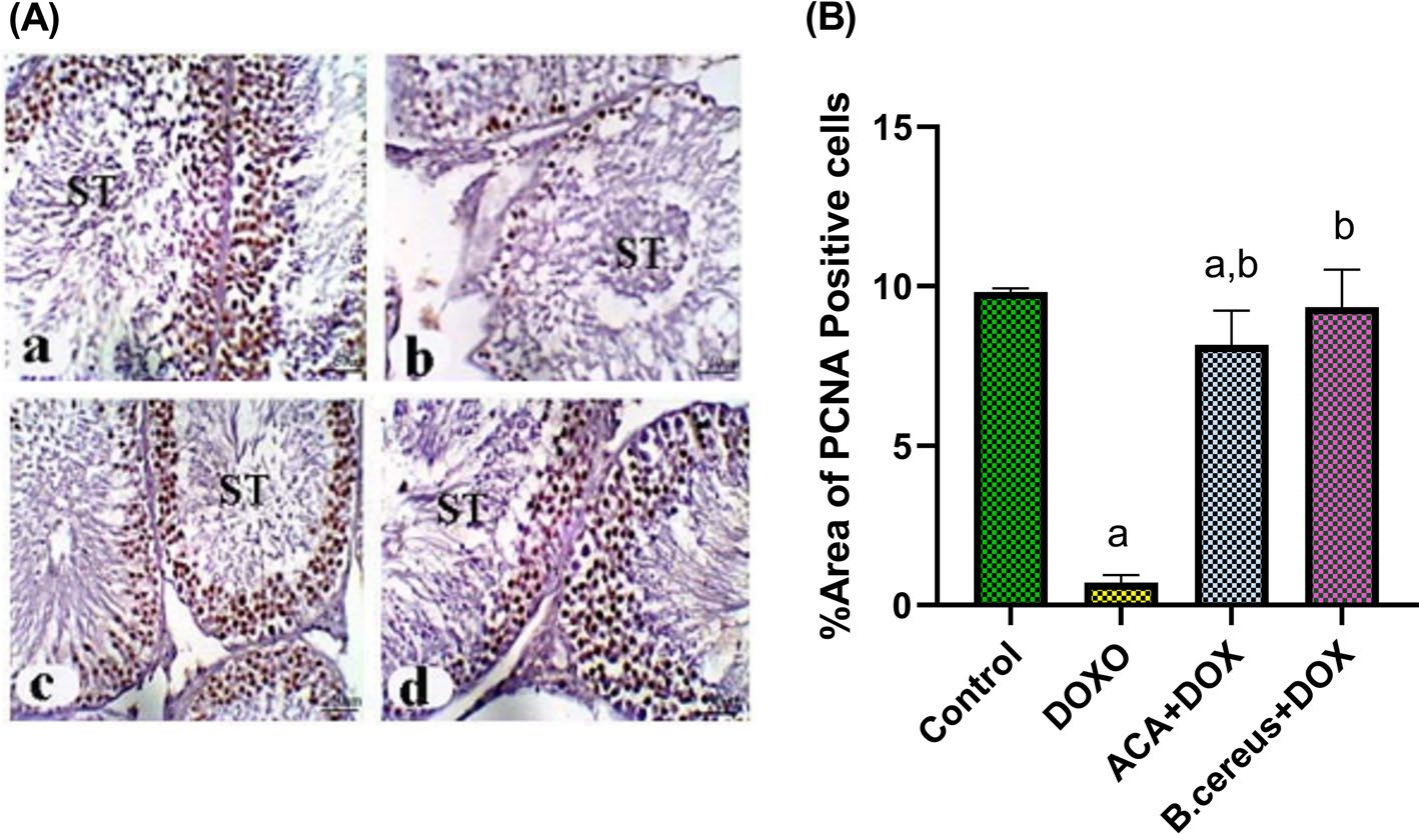
Immunohistochemical expression of PCNA in testis (**A**), and % area of PCNA positive cells (**B**) in DOX-induced testicular damage in rats: Control group (a); DOX-treated group (b); DOX + ACA–treated group (c); and DOX + *B. cereus* protease–treated group (d). X 200 (Scale bar = 50 μm). Each bar with a vertical line represents the mean ± S.D (*n* = 6). ^a^Compared with the control group, and ^b^compared with the DOX group. All values are statistically significant at *P* < 0.05. Abbreviations: ACA, acacetin; *B. cereus*, Bacillus cereus protease; DOX, doxorubicin; PCNA, proliferating cell nuclear antigen; ST, seminiferous tubules

Interestingly, immunohistochemical staining of testicular tissues from DOX-treated rats revealed that the most seminiferous tubules with negative PCNA expression, and a few seminiferous tubules with few PCNA immunoreactive spots in the nuclei of the spermatogenic cells with negative spermatocytes. In contrast, adminstration of ACA or *B. cereus* protease exhibited positive brown nuclei of spermatogonia and PCNA immunoreactive spermatocytes (Fig. 6A). This was evidenced by the ability of ACA to improve the reduction in the calculated area percentage of PCNA (*P* < 0.0001), whereas *B. cereus* restored PCNA to the normal (*P* = 0.75) (Fig. 6B).

### Testicular Histopathological Examination

**Fig. 7:**
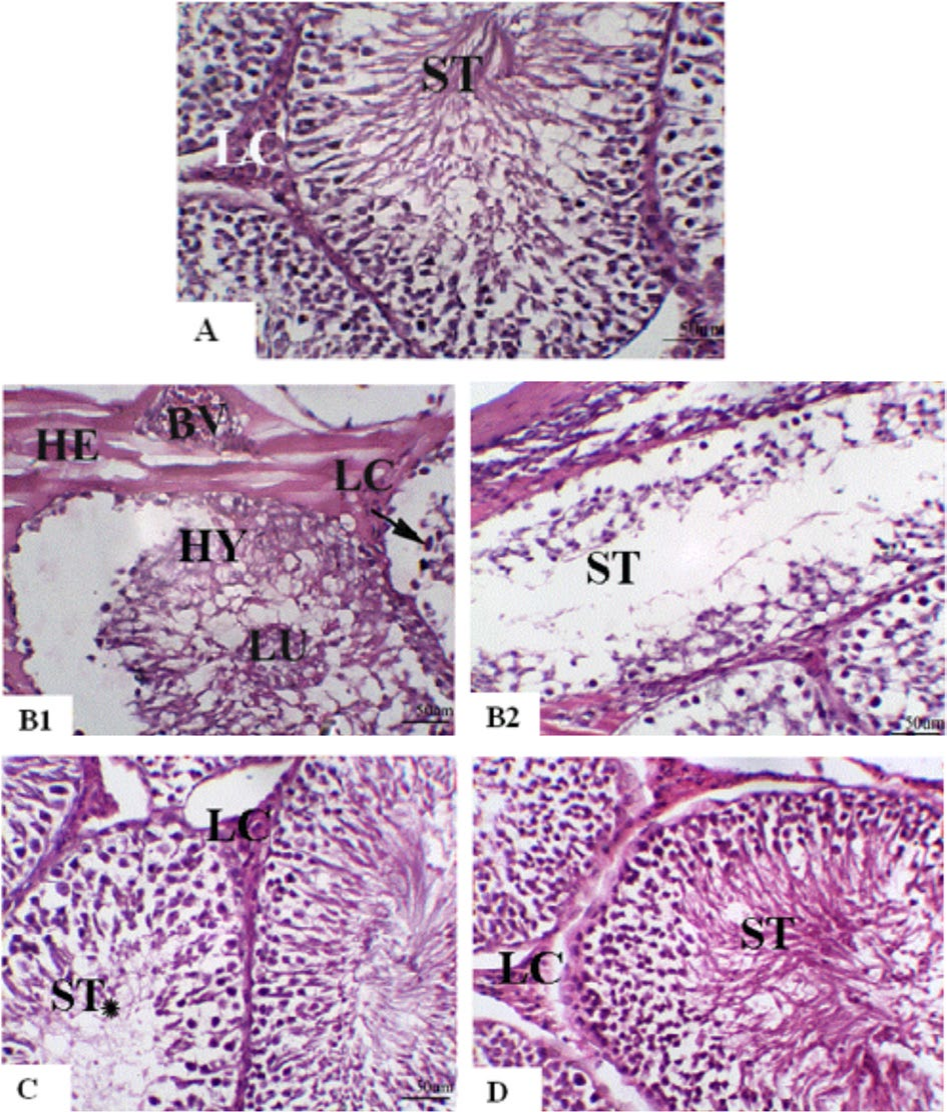
A photomicrograph of testis staining with H & E, X 200, (Scale bar = 50 μm). Control group (**A**), DOX-treated group (**B1** and **B2**), ACA + DOX**–**treated group (**C**), and DOX + *B. cereus* protease–treated group (**D**). Seminiferous tubules (ST); seminiferous tubules with a reduction in spermatogenic layers and complete absence of sperm (ST*); Leydig cells (LC); exfoliated spermatogenic cells in lumen (LU); hyaline degenerative changes (HY); pyknotic nuclei in the basal cell layers (arrow); hyalinizied congested blood vessels (BV); hemorrhage (HE)

Microscopic examination of testis sections stained with H&E from the control group revealed normal testicular architecture with complete normal spermatogenic layers and well-developed sperm (Fig. 7A). In contrast, DOX provoked marked pathological alterations compared with control. Most areas revealed scattered seminiferous tubules with loosely normal architecture, and hyaline degenerative changes accompanied by exfoliated spermatogenic cells in the lumen. In addition, many scattered pyknotic nuclei were observed in the basal cell layers of another seminiferous tubule. Marked widened interstitial areas with hemorrhage; thickened, hyalinized walls of the blood vessels; and a marked reduction in interstitial Leydig cells were apparent (Fig. 7B1). Other areas showed scattered seminiferous tubules with severe degenerative changes, and a prominent reduction in spermatogenic layers and sperm (Fig. 7B2) compared with the control group. However, pretreatment with ACA showed a moderate improvement in the form of a normal appearance of spermatogenic layers and sperm in most of the seminiferous tubules. However, a mild reduction in spermatogenic layers and a complete absence of sperm were occasionally observed in some seminiferous tubules. Mild widened interstitial spaces and moderated reductions in Leydig cells were also apparent compared with the DOX group (Fig. 7C). *B. cereus* protease pre-treatment group showed marked improvement as evidenced by no histological alterations in seminiferous tubules, complete spermatogenic layers, and a normal appearance of sperm and interstitial Leydig cells compared with the DOX-treated group (Fig. 7D).

It is worthy to be mentioned that *B. cereus* protease showed a stronger protective effect than ACA against DOX-induced testicular injury, as indicated by the protease’s capacity to restore the expression of Nrf2, TLR4, TNF-α, FOXO1, miR-155, caspase-3 levels, the Bax/Bcl2 ratio, and PCNA to the normal.

### Correlation Analysis

We found several pronounced correlations between parameters, as illustrated in.

Table 3. miR-34c showed a positive correlation with SIRT1, serum testosterone, and 17β-HSD levels, whereas it was negatively correlated with miR-155 and FOXO1. Alternatively, miR-155 exhibited a negative correlation with the aforementioned parameters and a positive correlation with FOXO1. SIRT1, serum testosterone levels, and 17β-HSD levels were positively correlated with one another and negatively correlated with FOXO1.

**Table 3:** Correlations between different biomarkers in all studied groups

	MiR-34c	MiR-155	SIRT1	FOXO1	Testosterone	17β-HSD
MiR-34c	1	− 0.706***	0.555**	− 0.798***	0.444*	0.499*
MiR-155	− 0.706***	1	− 0.794***	0.89***	− 0.771***	− 0.71***
SIRT1	0.555**	− 0.794***	1	− 0.914***	0.971***	0.966***
FOXO1	− 0.798***	0.89***	− 0.917***	1	− 0.862***	− 0.866***

Values represent Pearson correlation coefficient. Correlation is significant at *P < 0.05, **P < 0.01, and ***P < 0.001. *17β-HSD*, 17 beta-hydroxysteroid dehydrogenase; *FOXO1*, Forkhead box protein O1; *SIRT1*, sirtuin1; *miR*, micro RNA

## Discussion

Testicular damage is one of the most serious side effects of DOX exposure that eventually leads to male infertility [7]. In the current study, we demonstrated a protective effect of ACA and *B. cereus* protease against DOX-induced male infertility in rats. Our findings also support the involvement of miR-155/SIRT1/FOXO1 signaling pathway in DOX-induced male sterility, indicating that modulation of this network is implicated in the protective effects of ACA and *B. cereus* protease.

In the study, DOX revealed variable pathological changes compared with the control. Histopathological results showed scattered seminiferous tubules with severe degenerative changes and a prominent reduction in spermatogenic layers and sperm count compared with the control group. In parallel, DOX also caused a marked decline in serum testosterone levels. These results are consistent with that of Rizk et al. [56] who reported that DOX evoked a significant decrease in serum testosterone levels which have an impact on spermatogenesis as well as on the structural morphology of seminiferous tubules. Consistent with our findings, they also reported that DOX significantly dampened the activity of 17β-HSD which is the principal enzyme in the synthesis of male sex hormone. Nevertheless, pre-administration with ACA or *B. cereus* protease rescued the histological features and toxic effects of DOX on androgenic hormone synthesis. This was evident by the improved H&E staining picture along with a marked increase in serum testosterone levels and 17β-HSD activity. Intriguingly, the amelioration with the protease isolated from *B. cereus* was superior to that with ACA.

miR-34c is specifically expressed in germ cells. Bouhallier et al. [14] observed the highest expression of miR-34c in the testis, lower in the lungs, and virtually no expression in other organs. Moreover, they suggested that miR-34c expression is directly associated with germ cell numbers. Others showed that miR-34c is downregulated in the cryptorchidism model in mice [16]. Moreover, miR-34c downregulation in prostate cancer suppresses tumor migration and invasion [57]. Similar to previous studies, our findings revealed a significant downregulation in miR-34c expression in DOX-intoxicated rats compared with the normal which was reversed by pre-administration of ACA or *B. cereus* protease demonstrated its potential to protect against DOX-triggered male reproductive degeneration. Surprisingly, this protease was more effective than ACA.

Nrf2 is a transcriptional factor that plays a fundamental role in antioxidant defense mechanisms in various body tissues. It mitigates the damage of DOX, possibly through stimulation of antioxidant defense systems along with suppression of DOX-induced fibrotic and inflammatory responses [6]. The role of Nrf2 in protection against DOX-induced testicular damage was confirmed by our data which showed that the addition of ACA or the protease boosted Nrf2 content and was associated with a significant increase in antioxidant enzyme activity. Wu et al. [1] demonstrated that Nrf2 is important in mediating the protective effects of ACA against DOX cardiotoxicity, which in turn boosts antioxidant mechanisms, possibly through AMPK activation. In the same context, Cavello et al. [58] reported the antioxidant potential of a protease from bacteria Bacillus cytotoxicus.

GSH and SOD are nonenzymatic and enzymatic antioxidants that play a crucial role in reactive oxygen species (ROS) scavenging. Herein, as a result of DOX administration, the levels of these endogenous antioxidants decreased significantly, which may result from increased production of toxic DOX metabolites or reduced production of antioxidant defense systems [59], which results in oxidative stress. ACA and *B. cereus* protease caused a significant elevation in GSH level and SOD activity, which is consistent with a study by Wu et al. [1] who reported the antioxidant potential of ACA against DOX cardiotoxicity in cultured rat cardiomyoblasts and by others who examined the antioxidant defense of proteases of *Bacillu*s spp. [58, 60], indicating the antioxidant activity of ACA and *B. cereus* protease protects against DOX-induced testicular damage. According to Uygur et al. [61], DOX-induced DNA damage enhances the formation of ROS, causing a marked deterioration of testicular function. In the present study, we showed that the administration of DOX triggers oxidative stress and testicular lipid peroxidation. These findings are consistent with those of previous studies that demonstrated severe pathologic alterations in testicular tissue are linked to a high degree of lipid peroxidation [62, 63]. Increased MDA levels in the DOX group may be related to the deteriorating changes in the testes, which may be linked to the male germ cell membrane that contains an abundance of polyunsaturated fatty acids (PUFA) thus rendering the testes susceptible to lipid peroxidation [64]. These effects were ameliorated upon treatment with ACA, which concurred with the result of Wu et al. [65] who demonstrated a significant reduction in MDA levels with different concentrations of ACA, and that of Shiravi et al. [66] who reported that ACA inhibited renal MDA levels and elevated TAC in an ischemic reperfusion rat kidney model. In addition, *B. cereus* protease counteracted the increased production of ROS and lipid peroxidation, which is consistent with the results of Manivasagan et al. [67] who demonstrated the antioxidant effects of protease from *Streptomyces* spp.

NO is a reactive nitrogen species that contributes significantly to nitrosative stress. In the present study, we found that a significant increase in testicular NO levels in the DOX-treated group contributed to increased nitrosative stress. This may result from reduced SOD activity, which increased the availability of superoxide anion radicals, which then reacted with available NO to produce peroxynitrite, a cytotoxic agent and powerful radical [68]. On the other hand, pre-administration of ACA or the protease resulted in reduced NO overproduction and hence reduced nitrosative strain.

Oxidative stress is frequently associated with inflammation, as ROS can trigger pro-inflammatory transcription factors [69]. Our results indicated upregulation of the pro-inflammatory transcription factors TLR4, NF-κB, and TNF-α which coincides with previous studies demonstrating that DOX upregulated NF-κB and enhances pro-inflammatory markers in the heart [69] and testis [68]. Additionally, our findings are in line with others [35, 70] who demonstrated the potential of proteases and ACA to reduce and alleviate inflammation, suggesting the anti-inflammatory potential of both regimens.

SIRT1, is a deacetylase for many transcription factors, including FOXO1, and regulates several cellular processes, such as proliferation, differentiation, and apoptosis. miR-155 is a direct target of SIRT1 as miR-155 downregulated SIRT1 through SIRT1 3′ UTR binding. The current results indicate that increased TNF-α causes significant elevation of miR-155 expression, resulting in SIRT1 protein suppression in the DOX-treated group. These findings are in harmony with that of Guo et al. [71] who demonstrated that TNF-α significantly upregulated miR-155 expression which subsequently reduced SIRT1 expression. However, administration of ACA or *B. cereus* protease dampened the expression of miR-155 which may contribute to a reduced TNF-α induced SIRT1 suppression, suggesting an anti-inflammatory effect of ACA and *B. cereus* protease against DOX-induced testicular insults in rats via suppression of miR-155 and promotion of SIRT1 expression.

The FOXO protein family is primarily controlled by post-translational modification, including phosphorylation, and acetylation [72]. FOXO1 is expressed in many cell types and tissues throughout development, including endothelial, smooth muscle, neural crest, and male germ cells [72]. Tothova and Gilliland [73] identified FOXO1 as a requirement for spermatogenesis. Changes in FOXO1 expression result in spermatogenetic failure [28, 74]; however, there have not been sufficient studies showing a functional role for FOXO1 in DOX-induced testicular dysfunction. FOXO1 is a potential target of SIRT1 because SIRT1 directly inhibits the expression of FOXO1via deacetylation [75, 76]. Our findings revealed that DOX-induced SIRT1 downregulation by miR-155 upregulation results in acetylation and activation of FOXO1 triggered apoptosis, suggesting a regulatory role for FOXO1 in DOX-induced testicular apoptosis. However, the data showed downregulation of FOXO1 expression following treatment with ACA or *B. cereus*, which may result from SIRT1 suppressing FOXO1-induced cell apoptosis through deacetylation. The current data provide experimental evidence that miR-155 promotes testicular apoptosis by modulating SIRT1-dependant FOXO1 acetylation during DOX-induced testicular injury. In addition, we provide a new therapeutic approach using ACA and *B. cereus* protease to prevent DOX-induced testicular degeneration by modulating miR-155/SIRT1/FOXO1 network.

The expression of PCNA in spermatogonia and early phase primary spermatocytes at all stages in the seminiferous tubules occurs in testicular tissues. Because spermatogonia differentiation is a vulnerable step in the spermatogenic process, various chemicals can reduce the number of these cells [77]. In the present study, PCNA-positive cells were strongly detected in the spermatogonia of control rats. However, the number of PCNA-positive cells was considerably lower in the DOX-treated group. This observation is in harmony with other studies that reported DOX treatment is known to induce a reduction in PCNA in testicular germ cells, indicating a reduction in proliferating activity and spermatogenesis [78, 79]. DOX treatment is known to induce cell cycle arrest and death in replicating somatic cells [79]. In contrast, there was an increase in testicular PCNA expression in the ACA or *B. cereus* group compared with the DOX-treated group.

The Bcl-2 family regulates the apoptotic pathway and includes the pro-apoptotic Bax and BH3 subfamily (also known as BH3-only protein) and anti-apoptotic Bcl-2 subfamily [80]. Bim (Bcl-2 interacting mediator of cell death), one of the BH3-only proteins, is a FOXOs downstream target gene that interacts with Bax/Bcl-2, thus activating the Bax-induced mitochondrial pathway [81, 82]. Yao et al. [83] showed that Bim expression was significantly increased in H2O2-treated cells and reduced in cells with SIRT1 overexpression, indicating that SIRT1 inhibits Bim expression by regulating FOXO proteins. Together with these results, we found that an elevation of the testis apoptotic Bax/Bcl2 ratio in DOX-intoxicated rats was ameliorated upon pretreatment with ACA or the protease. This suggests a role for both prophylactic regimens on modulating FOXO-induced apoptosis. In the same context, high caspase-3 expression was detected in DOX-treated testicular cells, which agrees with the study of Tacar and Dass [84]. However, ACA or the protease-pretreated group exhibited lower expression levels of testicular caspase-3, indicating that both pretreatment regimens can alleviate the apoptotic signaling cascade pathway induced by DOX. These results suggest that DOX-impaired rat testicular architecture and spermatogenesis induce cell apoptosis, whereas ACA or *B. cereus* pre-treatment effectively protects against testicular apoptosis. This indicates a role for these compounds as novel therapeutics for the management of reproductive injury associated with DOX exposure.

## Conclusion

In the current study, we demonstrate for the first time that ACA or *B. cereus* protease are potential therapeutic agents that offer protection against the detrimental effects of DOX on the male reproductive system through modulation of miR-155/SIRT1/FOXO1 signaling. This treatment regimen may improve the quality of life and self-image of men.

## Supplementary Information

Below is the link to the electronic supplementary material.

Supplementary file1 (DOCX 17 KB)

## Data Availability

The data and materials are included in this published article.

## Abbreviations

17β-HSD: 17β-Hydroxysteroid dehydrogenase
ACA: Acacetin
ANOVA: One‐way analysis of variance
*B. cereus* protease: Bacillus cereus protease
Bax: Bcl-2-associated X protein
Bcl2: B-cell lymphoma 2
DMSO: Dimethyl sulfoxide
DOX: Doxorubicin
ELISA: Enzyme-linked immunoassay
FOXO1: Forkhead box protein O1
GSH: Reduced glutathione
H&E: Hematoxylin–eosin, i.p intraperitoneal
MDA: Malondialdehyde
miR-155: MicroRNA-155
miR-34c: MicroRNA-34c
miRNAs: MicroRNAs
NF-κB: Nuclear factor kappa-light-chain-enhancer of activated B cells
NO: Nitric oxide
Nrf2: The nuclear factor erythroid 2-related factor 2
PCNA: Proliferating cell nuclear antigen, p.o peroral
S.D: Standard deviation
SIRT1: Sirtuin-1
SOD: Superoxide dismutase
TAC: Total antioxidant capacity
TLR4: Toll-like receptor 4
TNF-α: Tumor necrosis factor alpha
