# Supplementary material for: Inflammatory Modulation of miR-155 Inhibits Doxorubicin-Induced Testicular Dysfunction via SIRT1/FOXO1 Pathway: Insight into the Role of Acacetin and Bacillus cereus Protease
Source: Appl Biochem Biotechnol. 2022 Jun 18;194(11):5196–219. doi: 10.1007/s12010-022-03992-8 (PMC9587093; doi:10.1007/s12010-022-03992-8)
Supplement: Supplementary file 1 — Supplementary file1 (DOCX 17 KB) [file 12010_2022_3992_MOESM1_ESM.docx]

**Supplementary material table:** The body and testis weights of different groups.

| **Control** | | **DOX** | | **ACA + DOX** | | **B. cereus + DOX** | |
| --- | --- | --- | --- | --- | --- | --- | --- |
| Body weight | Testis weight | Body weight | Testis weight | Body weight | Testis weight | Body weight | Testis weight |
| 170 | 1.73 | 136 | 0.94 | 139 | 1.4 | 150 | 1.45 |
| 165 | 1.53 | 129 | 1.1 | 142 | 1.38 | 149 | 1.58 |
| 166 | 1.67 | 132 | 1.02 | 141 | 1.34 | 146 | 1.39 |
| 170 | 1.72 | 134 | 0.96 | 143 | 1.37 | 149 | 1.58 |
| 165 | 1.53 | 129 | 1 | 139 | 1.4 | 150 | 1.45 |
| 167 | 1.66 | 132 | 1.03 | 141 | 1.34 | 146 | 1.4 |
